# Biological insights into the role of TET2 in T cell lymphomas

**DOI:** 10.3389/fonc.2023.1199108

**Published:** 2023-09-29

**Authors:** Shannon A. Carty

**Affiliations:** Division of Hematology-Oncology, Department of Internal Medicine, University of Michigan, Ann Arbor, MI, United States

**Keywords:** TET2, angioimmunoblastic T cell lymphoma, T follicular helper cell lymphoma, epigenetic therapy, peripheral T cell lymphoma (PTCL)

## Abstract

Peripheral T cell lymphomas (PTCL) are a heterogenous group of mature T cell lymphomas with an overall poor prognosis. Understanding the molecular heterogeneity in PTCL subtypes may lead to improved understanding of the underlying biological mechanisms driving these diseases. Mutations in the epigenetic regulator TET2 are among the most frequent mutations identified in PTCL, with the highest frequency in angioimmunoblastic T cell lymphomas and other nodal T follicular helper (TFH) lymphomas. This review dissects the role of TET2 in nodal TFH cell lymphomas with a focus on emerging biological insights into the molecular mechanism promoting lymphomagenesis and the potential for epigenetic therapies to improve clinical outcomes.

## Introduction

Peripheral T cell lymphomas (PTCL) are a heterogenous group of aggressive lymphomas derived from mature T cells and account for 10-15% of all non-Hodgkin lymphomas (1). Among PTCL cases, the World Health Organization (WHO) classification has recently recognized a distinct entity termed nodal T follicular helper cell (TFH) lymphomas, which include the subtypes previously termed angioimmunoblastic T cell lymphoma (AITL), follicular T cell lymphoma and peripheral T cell lymphoma with a TFH phenotype (2). Nodal TFH lymphomas share phenotypic and gene expression similarities with normal T follicular helper (TFH) cells (3–5), a CD4^+^ T cell subset that promotes germinal center B cell differentiation (6). In the International Peripheral T-cell and Natural Killer/T-cell Lymphoma study, AITL (at the time the most recognized nodal TFH lymphoma) accounted for approximately 20% of PTCL cases and thus is the second most common PTCL subtype after PTCL, not otherwise specified (1). Clinically, nodal TFH lymphomas typically present at an advanced stage with lymphadenopathy, hepatosplenomegaly and constitutional symptoms, as well as various autoimmune manifestations (7–9). PTCLs, including nodal TFH lymphomas, have an overall poor prognosis with AITL patients having an expected 5-year overall survival of ~30% (1, 10). Recurrent somatic mutations in multiple epigenetic regulators, including loss-of-function mutations in TET2, inactivating mutations in DNMT3A and neomorphic mutations in IDH2, have been strongly associated with nodal TFH lymphomas (11–16). Given the poor prognosis of these lymphomas, understanding the underlying biology is critical to design therapies with improved efficacy. Given that TET2 is the most commonly mutated epigenetic regulator in these lymphomas, this review will focus on the mechanistic role of TET2 in the development and treatment of nodal TFH lymphomas.

## TET2 function

TET2 is a member of the ten-eleven-translocation (TET) family of Fe^2+^- and alpha-ketoglutarate-dependent methylcytosine dioxygenases. These enzymes oxidize 5-methylcytosine (5mC) to 5-hydroxymethylcytosine (5hmC) and subsequent oxidized methylcytosine intermediates to ultimately generate an unmodified cytosine (17–19). DNA methylation was long thought to be a relatively stable epigenetic mark; however, the discovery of the TET family of enzymes introduced the concept of active DNA demethylation. TET2 is broadly expressed in hematopoietic cells and TET2 loss promotes hematopoietic stem cell (HSC) and myeloid cell expansion in murine models (19–22). Studies of TET2 function in murine and human hematopoietic cells reveal that TET2 deletion or loss-of-function mutations, such as those in nodal TFH lymphomas, lead to altered DNA methylation and chromatin accessibility at regulatory enhancer regions (23–25), suggesting functional epigenetic consequences.

In the study of TET2 function in T cells, deletion of TET2 in mature T cells does not result in any appreciable alteration in late T cell development or peripheral T cell activation (26). However, in antigen-specific CD4^+^ T cells, TET2 loss leads to an increase in TFH differentiation in a cell-intrinsic manner with hypermethylation at gene loci associated with helper T cell differentiation (27), suggesting that TET2 directly represses TFH differentiation by demethylating key regulatory loci.

## TET2 and associated mutations in T cell lymphomas

Loss-of-function mutations in TET2 were first identified in myeloid malignancies (28, 29) but soon thereafter recurrent somatic mutations in TET2 were recognized in approximately 50-70% of AITL and other TFH-derived lymphomas (11–15). Subsequent gene sequencing of other T cell leukemias/lymphomas revealed TET2 mutations at much lower frequencies compared to nodal TFH lymphomas – including 17% of T-cell prolymphocytic leukemia cases (30), 14-20% of acute T-cell leukemia/lymphoma cases (31, 32) and in 4-12% cases of cutaneous T cell lymphoma and/or Sezary syndrome cases (33–35).

Furthermore, frequent mutations at isocitrate dehydrogenase 2 arginine 172 (IDH2 R172) have also been identified in AITL and other TFH-derived lymphomas (16). IDH2 is a mitochondrial enzyme that typically converts isocitrate to 2-alpha-ketoglutarate (aKG); however, the R172 mutation promotes abnormal oncometabolite production of the R-enantiomer of alpha-hydroxyglutarate (aHG) (36), which competitively inhibits aKG-dependent enzymes including the TET family (37). Thus, nodal TFH-derived lymphomas with IDH2 R172 mutations are predicted to have repressed TET activity and accordingly IDH2-mutated AITL exhibits genome-wide DNA hypermethylation compared to IDH2 wild-type AITL (38).

## TET2 role in lymphomagenesis

Despite frequent TET2 mutations in a wide array of T cell lymphomas, most commonly in nodal TFH lymphomas, it was initially unclear the degree to which TET2 loss-of-function directly contributed to lymphomagenesis. TET2 deletion in murine hematopoietic stem cells (HSCs) altered early and late hematopoiesis in both myeloid and lymphoid lineages with eventual development of myeloid malignancies in the mice (14, 21, 22) but only rarely mature lymphoid malignancies (14). A murine model with a hypomorphic TET2 allele does develop TFH-like lymphomas but with a prolonged latency (39).

In several sequencing studies of nodal TFH lymphomas including AITL, multiple TET2 mutations were found in individual tumor samples implying a strong selective pressure (12, 13, 40). Additionally, in AITL cases, the majority of the cases that carried a TET2 mutation had a variant allele frequency >10% (12, 38, 40).

Since TET2 mutations were frequently found to co-occur with a glycine to valine (G17V) inactivating mutation in Rho GTPase RhoA in 50-70% of AITL cases (12, 40, 41), several groups sought to dissect the relative contribution of RhoA-G17V mutations and TET2 loss of function to T cell lymphomagenesis. Adoptive transfer of wild-type or TET2-deficient T cells retrovirally transduced to overexpress RhoA-G17V into T-cell deficient murine hosts resulted in CD4^+^ T cell expansion, disruption of peripheral T cell homeostasis and eventually lethal inflammation but no lymphoma was noted (42). Several other groups generated transgenic mice expressing the RhoA-G17V mutation in the setting of TET2 hematopoietic deficiency. In these various murine models, RhoA-G17V overexpression in T cells promoted TFH proliferation/expansion (43, 44) and the concomitant expression in the setting of hematopoietic TET2 deficiency led to the development of TFH lymphomas with varying penetrance (43–45). On a molecular level, RhoA-G17V and TET2 loss was found to promote mammalian target of rapamycin complex 1 (mTORC1) pathway activation (43, 44) and inactivation of forkhead box O1 (FOXO1) signaling (44), suggesting potential therapeutic targets. Together these data strongly support a role for RhoA-G17V as a driving mutation in nodal TFH lymphomas but also speak to the requirement for concomitant TET2 loss in the hematopoietic compartment to promote lymphomagenesis. Targeting of downstream pathways, such as with mTOR inhibitors, may be an attractive therapeutic target to be tested in nodal TFH lymphomas, though no trials are currently underway.

## TET2 in clonal hematopoiesis and tumor microenvironment

Mutations in TET2 are among the three most frequent somatic mutations in age-related clonal hematopoiesis and are associated with an increased risk of hematologic cancers as well as all-cause mortality (46–48). The extent to which TET2 mutations in the lymphoma microenvironment and responding immune cells contributes to T cell lymphomagenesis has not been fully elucidated.

It has been posited that TET2 mutations noted in nodal TFH lymphomas largely arise in the setting of clonal hematopoiesis, which is supported by the fact that TET2 mutations in T cell lymphoma patients are frequently found to co-occur in the non-neoplastic B lymphocyte, myeloid and HSC compartments as well as the neoplastic T cells (14, 49, 50). In patients with AITL, the majority of patients had TET2 mutations identified in the neoplastic T cells as well as the myeloid compartment (51). In this case series, 4 of 22 patients with TET2 mutations and available sequencing data developed myeloid neoplasms approximately 2-4 years following their lymphoma diagnosis. The myeloid neoplasms all shared multiple TET2 mutations in the myeloid clone and AITL cells but also contained additional different mutations that were not shared. Together these data support myeloid neoplasms arising from early clonal TET2-mutated hematopoietic stem cells but with divergent evolution from the neoplastic AITL cells.

The presence of TET2-mutated immune cells in AITL patients led to the question if TET2 mutations alter tumor immunity to promote T cell lymphomagenesis. TET2 is known to have pleiotropic functions in different immune cells known to play a role in tumor immunity, including macrophages/monocytes, CD4^+^ helper T cells, T regulatory cells, CD8^+^ T cells and B cells (52). In myeloid cells, TET2 represses inflammatory gene expression (53) with increased IL-6, IL-1β and arginase 1 in TET2-deficient macrophages (54, 55). In a murine melanoma model, TET2 deletion in myeloid cells resulted in reduced tumor burden and increased tumor-infiltrating T cells suggesting that TET2 promotes a myeloid immunosuppressive program in the tumor microenvironment (56). In CD4^+^ T cells, TET2 inhibits cytokine production, including IFNγ, IL-17 and IL-10 (57), cytokines which can have both immunostimulatory and immunosuppressive roles. Furthermore, TET2 (in combination with either TET1 or TET3) dampens regulatory T cell immunosuppressive function (58, 59), which are a critical cellular subset known to suppress anti-tumor responses (60). In CD8^+^ T cells, TET2 represses memory differentiation following infection (26), though less is known about the role of TET2 deficiency in CD8^+^ T cell anti-tumor immunity and T cell exhaustion. Given these pleotropic roles TET2 may play in the tumor microenvironment, it is important to carefully analyze the tumor-intrinsic versus microenvironmental roles TET2 loss-of-function mutations play in promoting nodal TFH lymphomas.

A recent elegant study dissected T cell-intrinsic versus -extrinsic role of TET2 in lymphomagenesis using murine models with either hematopoietic or T cell specific loss of TET2 crossed to RhoA-G17V transgenic mice (61). TET2 deficiency in all hematopoietic cells accelerated the development of TFH lymphomas compared to either a wild-type hematopoietic compartment or TET2 deletion solely in T cells. To test which immune compartment contributed to TFH lymphomagenesis, the authors co-transplanted tumor cells with a variety of immune lineages into immunodeficient mice and monitored tumor development. Only when B cells were co-transplanted did donor-derived tumors develop suggesting that TET2 loss in B cells supported TFH lymphomagenesis. Subsequent analysis revealed clonal expansion of TET2-deficient germinal center B cells in the tumor-bearing mice, unique mutations in core histones developed in murine clonal B cells and that inhibition of CD40-CD40L interactions prolonged survival in mice. Correlative studies in human AITL samples demonstrated an expansion of germinal center B cells in involved lymph nodes and unique mutations (some also in core histone genes) in the tumor-associated B cells and plasma cells. These data strongly support a cooperating role for TET2-mutated B cells in the immune microenvironment to promote nodal TFH lymphoma development. Targeting these interactions could provide a novel therapeutic avenue in nodal TFH lymphoma patients, although it remains unclear if this mechanism occurs outside of TET2-mutated clonal hematopoiesis.

## Treatment and prognosis implications

Since AITL and other nodal TFH lymphomas have an overall poor prognosis with currently available treatments (1, 10), novel therapeutic approaches are needed to improve patient outcomes. TET2 mutations have been noted to be associated with adverse clinical parameters (13, 62) but not associated with a change in overall survival (13). Given the frequency of TET2 and other epigenetic mutations (ie, DNMT3A) that occur in the majority of nodal TFH lymphomas, there is great interest in utilizing epigenetic therapies to target underlying biological mechanism in hopes to improve response rates and survival. PTCL has been shown to be uniquely responsive to one type of epigenetic therapy, specifically histone deacetylase (HDAC) inhibitors, with three HDAC inhibitors approved for systemic PTCL: romidepson, belinostat and chidamide (in China). In the phase II trial of romidepsin in relapsed/refractory PTCL, patients with relapsed/refractory AITL had an overall response rate of 33% compared to 25% of the overall cohort with two-thirds of the AITL responders achieving a complete remission (63). Similarly, in the phase II registration study of belinostat in relapsed/refractory PTCL, patients with AITL seemed to have improved response rates (45%) compared to response rate (26%) of the overall trial population (64). A more recent retrospective, multicenter study comparing HDAC inhibitor responses in TFH versus non-TFH PTCL patients found a significantly improved overall response rate in nodal TFH *vs.* non-TFH lymphomas (56.5% versus 29.4%) (65). Together these data support the idea that nodal TFH lymphomas may be more sensitive to epigenetic modulation than non-TFH lymphomas (summarized in **Table 1**), whether this sensitivity correlates with the presence of epigenetic alterations due to TET2 mutations remains unknown.

**Table 1:** Response rates in epigenetic therapies in relapsed/refractory nodal TFH lymphomas versus overall PTCL.

Study	Type	Disease Status	Overall		TFH/AITL	
			Number	ORR	Number	ORR
Romidepsin (54)	Phase II	R/R	130	25%	27	33%
Belinostat (55)	Phase II	R/R	129	26%	22	45.5%
HDAC inhibitor (56)	Retrospective	R/R	127	45.6%	76	56.5%
Aza/Romidepsin (63)	Phase II	Tx Naïve & R/R	23	61%	15	80%

ORR, overall response rate; R/R, relapsed/refractory; Tx, treatment.

Given TET2’s function in active DNA demethylation, questions naturally arise about the role of hypomethylating agents (HMAs) in nodal TFH lymphomas. Several case reports and case series suggest some clinical efficacy of single agent HMAs (5-azacitidine or decitabine) in TET2-mutated angioimmunoblastic T cell lymphoma (66–69). Preclinical studies have suggested synergy between HMAs and HDAC inhibitors in T cell lymphomas (70, 71) providing a biologic rationale for combined epigenetic targeted therapy in PTCL patients. A multicenter phase II trial examining the combination of oral 5-azacitidine and romidepsin in treatment naïve and relapsed/refractory PTCL patients found that patients with a TFH phenotype had higher overall response rate (80%) and complete response (60%) compared to the overall response rate (25%) and complete remission rate (12.5%) among patients with other subtypes (72). In this early-phase study, there were no statistical differences in response rates between patients with wild-type or mutated TET2 but this was limited by small sample size. Together these data suggest that duel epigenetic targeting therapies may be particularly effective in nodal TFH lymphomas.

Since patients with relapsed/refractory AITL have progressively shorter remissions with each subsequent line of therapy (73), the best chance to cure patients likely lies in improving first-line therapies. Based on the emerging understanding of the underlying biology and the role of epigenetic targeted therapies in nodal TFH lymphomas, several studies have been undertaken to combine epigenetic therapy with standard front-line chemotherapy (CHOP, cyclophosphamide, doxorubicin, vincristine and prednisone). A randomized phase III trial compared to romidepsin plus CHOP to CHOP alone in treatment naïve patients with PTCL. Unfortunately, there were no differences in response rates, progression free survival or overall survival and there were more treatment-related adverse advents in the Ro-CHOP arm (74). However, in an exploratory analysis, PTCL patients with a TFH phenotype had improved progression free survival after Ro-CHOP compared CHOP suggesting that nodal TFH lymphomas may derive a unique benefit and clinical trials should be further focused on PTCL subsets. Clinical trials focused on nodal TFH PTCL populations are examining the efficacy of combining azacitidine (NCT03542266) or chidamide (NCT03853044) with frontline CHOP. Recently published results of the phase II trial of oral azacitidine plus CHOP in 20 evaluable PTCL patients demonstrated a complete response in 88.2% of PTCL-TFH patients and 2-year progression free survival of 69.2% in PTCL-TFH patients. Notably, TET2 mutations were significantly associated with complete response rates and overall survival (75). The oral azacitidine plus CHOP combination is being tested in an ongoing randomized phase II trial in previously untreated patients with CD30-negative PTCL (NCT04803201).

## Conclusions

From the initial identification of TET2 mutations in AITL and other nodal TFH lymphomas just over twenty years ago, significant strides have been made to advance the understanding of TET2’s role in the pathogenesis of these lymphomas. Namely, TET2 loss of function in the lymphoma microenvironment, which arises in the setting of clonal hematopoiesis, likely play a critical role in supporting TFH transformation and AITL development. Additionally, emerging clinical evidence suggests that epigenetic targeted therapies may improve response rates and survival in patients with nodal TFH lymphomas. Using the evolving scientific knowledge about the underlying biology of these rare lymphomas, future clinical trials may need to tailor trial populations to discern true efficacy of these therapies.

## Author contributions 

The author reviewed the literature, performed the writing and revisions of the manuscript.
